# Advancing diagnostics and therapy in transthyretin amyloid cardiomyopathy

**DOI:** 10.1002/ehf2.15166

**Published:** 2024-11-26

**Authors:** Katarzyna Holcman, Michał Tkaczyszyn

**Affiliations:** ^1^ Department of Cardiac and Vascular Diseases Jagiellonian University Medical College, St. John Paul II Hospital Krakow Poland; ^2^ Department of Nuclear Medicine St. John Paul II Hospital Krakow Poland; ^3^ Institute of Heart Diseases Wroclaw Medical University Wrocław Poland; ^4^ Institute of Heart Diseases University Hospital Wrocław Poland

Transthyretin amyloid cardiomyopathy (ATTR‐CM) is a progressive and life‐threatening cardiac disease caused by the extracellular deposition of misfolded transthyretin (TTR) protein fibrils.^1^ The condition manifests in two primary forms: hereditary ATTR‐CM, which is associated with pathogenic variants in the TTR gene, and wild‐type ATTR‐CM (wtATTR‐CM), which develops due to age‐related structural and conformational abnormalities regarding TTR protein.^2^ The clinical presentation of ATTR‐CM at the time of diagnosis is variable, ranging from asymptomatic cardiac involvement [detected in cardiac magnetic resonance (CMR) imaging] to advanced heart failure (HF) resistant to conventional therapies.^3^, ^4^ Myocardial pathology is often accompanied by diverse systemic manifestations such as carpal tunnel syndrome, peripheral neuropathy and autonomic dysfunction, to name but a few.^5^ There are data demonstrating that certain combinations of clinical characteristics can improve the identification of this specific cardiomyopathy aetiology in large cohorts of patients.^6^ The care and therapy of patients with ATTR‐CM generate excessive costs for healthcare systems, higher than the population of HF patients without ATTR‐CM.^7^ Despite the increasingly available modern therapies modifying the natural history of the disease, the prognosis in ATTR‐CM is still poor and mortality rates are high.^3^, ^8^ The diagnosis of this underlying cause of HF is frequently made late, leading to the introduction of targeted treatment at the stage of already advanced cardiac impairment and diminished quality of life.^4^, ^9^ Indeed, despite increasing awareness of ATTR‐CM among cardiologists (especially those managing HF patients), this condition remains underdiagnosed in clinical practice and is frequently mistaken for other aetiologies, such as sarcomeric hypertrophic cardiomyopathy (HCM). This is due to the substantial overlap in their phenotypic characteristics, including increased left ventricular (LV) wall thickness and diastolic dysfunction.^10^, ^11^

In the context of the evolving understanding of the diverse aetiologies contributing to the common clinical and echocardiographic presentation of HCM, the recent research of Garcia‐Pavia et al., published in the *ESC Heart Failure*, highlights the need to revisit our diagnostic framework to include a more targeted approach to identifying ATTR‐CM.^12^ The TTRACK was a multicentre, non‐interventional, cross‐sectional epidemiological study that comprehensively evaluated the prevalence and characteristics of ATTR‐CM among a cohort of patients with HCM of unknown aetiology. The study was conducted across 20 centres in 11 countries, yielding a total number of 766 patients. The inclusion criteria in the study were age ≥50 years, clinical diagnosis of HCM defined by maximal end‐diastolic LV wall thickness ≥15 mm in echocardiography, and lack of any identifiable genetic or alternative aetiologies of the aforementioned LV hypertrophy. Study participants underwent bone scintigraphy with or without single‐photon emission computed tomography (SPECT), the gold standard non‐invasive imaging technique for diagnosing ATTR‐CM. Cardiac uptake of the radiotracers was visually graded according to the Perugini scale (0–3; the higher the pathological tracer uptake, the higher the grade). Monoclonal protein testing and TTR gene sequencing were performed for patients with abnormal (grade 1–3) cardiac uptake, enabling the differentiation between ATTR‐CM and haematological disease with cardiac involvement (light‐chain amyloidosis).^12^ The study revealed that 27% of patients with HCM had moderate‐to‐high cardiac uptake (grade 2 or 3) on scintigraphy, and out of these, 144 patients (19% of the total cohort) were finally diagnosed with ATTR‐CM (after excluding monoclonal gammopathy; importantly, the vast majority had wtATTR‐CM). The high prevalence of ATTR‐CM within a population of HCM of unknown aetiology underscores the importance of routine screening in patients presenting with unexplained LV hypertrophy. A particularly noteworthy finding was the association between specific clinical characteristics and a higher likelihood of ATTR‐CM diagnosis. Carpal tunnel syndrome, a well‐documented ‘red flag’ for ATTR‐CM, emerged as the most strongly associated non‐cardiac characteristic, with an odds ratio of 54 (*P* < 0.0001).^12^ Male sex, older age and increased LV septal wall thickness were also identified as significant correlates of ATTR‐CM in multivariable analyses. It needs also to be acknowledged that many patients with grade 1 (low) cardiac uptake of the tracer on scintigraphy were classified as having an undetermined status.^12^ This observation introduces another clinically relevant question—how clinicians should manage and follow‐up these patients? Further research on longitudinal follow‐up of this subgroup is needed to determine whether these patients represent early‐stage ATTR‐CM and, if so, how progression to more advanced disease might be prevented and/or mitigated.

Constant development and increasing availability of non‐invasive imaging techniques have significantly improved the ability to differentiate ATTR‐CM from other cardiomyopathies presenting with increased LV wall thickness.^13^, ^14^ Scintigraphy using bone‐avid tracers, along with modern complementary analyses of standard transthoracic echocardiography and CMR imaging, have redefined ATTR‐CM from a rare, unknown disease to a commonly considered aetiology of left (and also right) ventricular hypertrophy and/or dysfunction in the differential diagnosis.^15^, ^16^, ^17^ This applies also to patients with aortic stenosis scheduled for transcatheter valve implantation or patients with HF with reduced ejection fraction (EF) who do not tolerate conventional therapies and frequently experience the worsening of primary disease.^18^, ^19^, ^20^ It needs to be acknowledged that accumulating evidence demonstrates significant prevalence (even several per cent) of ATTR‐CM in aforementioned specific sub‐groups of patients, especially HCM, HF with preserved EF or aortic stenosis.^8^, ^12^, ^16^, ^18^

A correct diagnosis made without unnecessary delay in patients with ATTR‐CM is a prelude to initiating therapies that modify the natural history of the disease, which are becoming more widely available despite the objectively high costs of therapy (e.g., tafamidis or ribonucleic acid interference agents). However, many questions remain regarding standard therapies recommended regardless of HF aetiology depending on the systolic (dys)function of LV.^21^ Patients with ATTR‐CM may, for example, have poorer tolerance of standard drugs used in HF such as beta‐blockers or angiotensin‐converting enzyme inhibitors, and the management of such intolerance is rather intuitive than empirical. Moreover, we know little about the effectiveness of interventional therapies specifically in HF with reduced EF due to ATTR‐CM, such as electrotherapy (e.g., response to cardiac resynchronization therapy or efficacy of cardioverter‐defibrillator therapy to prevent sudden cardiac death) or transcatheter repair procedures on mitral or tricuspid valves.^22^ Patients with ATTR‐CM also have comorbidities typical of other aetiologies of HF, such as atrial fibrillation or iron deficiency, that may, for example, require slightly different approaches.^23^, ^24^, ^25^

The advancements in non‐invasive diagnostic algorithms have revolutionized our approach to diagnosing ATTR‐CM, enabling earlier and more accurate detection that fundamentally alters patient management and outcomes. If not randomized studies, then at least analyses of available longitudinal data on the effectiveness of individual treatments and therapies in this group of patients, certainly underrepresented in classical clinical trials on HF (irrespective of EF), seem reasonable in order to respond to the challenges of everyday clinical practice for these patients. ATTR‐CM represents a unique aetiology of HF, where the myocardial pathology can be treated causally, thereby inhibiting the progression of the disease.

## Conflict of interest statement

K.H. reports honoraria for lectures and participation in advisory boards and/or drug clinical trials of Pfizer, IONIS, Alnylam Pharmaceuticals, Eidos Therapeutics, Bayer, AstraZeneca and Swedish Orphan Biovitrum. M.T. reports personal fees from Eidos Therapeutics and Alnylam Pharmaceuticals for the participation in clinical trials on cardiac amyloidosis (sub‐investigator).
